# Complete mitochondrial genome sequence of giant freshwater prawn, *Macrobrachium rosenbergii* of Bangladesh

**DOI:** 10.1128/mra.00924-24

**Published:** 2024-11-27

**Authors:** Dipta Chandra Pal, Md. Mahmud Hasan, Shakila Nargis Khan, Muhammad Manjurul Karim

**Affiliations:** 1 Department of Microbiology, University of Dhaka 95324 , Dhaka, Bangladesh; 2 Fisheries Biotechnology Division, National Institute of Biotechnology, Dhaka, Bangladesh; University of California Riverside, Riverside, California, USA

**Keywords:** *Macrobrachium rosenbergii*, giant freshwater prawn, mitochondrial genome

## Abstract

We report the complete mitochondrial genome sequence of the giant freshwater prawn (*Macrobrachium rosenbergii*) from Bangladesh. The circular genome is 15,766 base pairs long, with a GC content of 38.1%, and contains 13 protein-coding genes, 22 tRNA genes, 2 rRNA genes, and 1 control region.

## ANNOUNCEMENT

The giant freshwater prawn, *Macrobrachium rosenbergii*, commonly known as the Golda prawn in Bangladesh, is a species of high economic and nutritional value. In Bangladesh, particularly in the southwestern region, the cultivation of *M. rosenbergii* plays a significant role in the economy, generating an annual revenue of approximately US $500 million and contributing 3.78% to the country’s GDP (1).

Despite its economic importance, the mitochondrial genome sequence for the local *M. rosenbergii* population remains unexplored. Previous studies have mapped the mitochondrial genomes of *M. rosenbergii* populations from Australia (2), China (3, 4), and Indonesia (5). However, there is a lack of data on the mitochondrial genome of *M. rosenbergii* from Bangladesh. To address this gap, our study reports the complete mitochondrial genome sequence of *M. rosenbergii* from Bangladesh, which will provide valuable genetic insights for molecular phylogeny, evolutionary, biogeographic relationship, aquaculture management, and conservation studies.

For this study, a specimen of *M. rosenbergii* was collected from Bhola River, Bangladesh (22°41′12.9″N; 90°38′38.2″E). The entire prawn was immediately preserved at −26°C until DNA extraction. Crude mitochondria were isolated from fresh telson tissue (0.1 g) following the Wieckowski protocol with modifications (6). Mitochondrial DNA was then purified using the phenol/chloroform extraction method (7). NGS library preparation was performed using the Illumina DNA Prep kit (Illumina, San Diego, CA, USA), and sequencing was carried out on the Illumina NextSeq 550 platform, generating paired-end reads of 151 bp in length. A total of 2,220,076 raw reads were generated, corresponding to 317.4 Mbp of sequencing data. The sequenced genome had an average depth of coverage of 50×. The raw data quality was checked using FastQC version 0.12.1 (8), and low-quality reads (*Q* < 20) were filtered out using TrimGalore version 0.6.10 (9). The *de novo* genome assembly of the trimmed reads was conducted using SPAdes genome assembler version 3.15.5 (10), resulting in the generation of a 15,766-bp MT-genome contig. The assembled genome showed 99.52%, 98.92%, and 93.23% identity to previously identified circular mitochondrial genomes of *M. rosenbergii,* with NCBI accession numbers ON783028.1, KY865098.1, and NC_006880.1, respectively. Gene annotation was performed using MITOS version 1.1.7 with the Invertebrate Mitochondrial Code (transl_table = 5) (11). Additionally, the precise gene regions were refined through manual curation based on NCBI nucleotide BLAST results against mitochondrial reference genomes of *M. rosenbergii* (NC_006880.1, KY865098.1, and ON783028.1).

**Fig 1:**
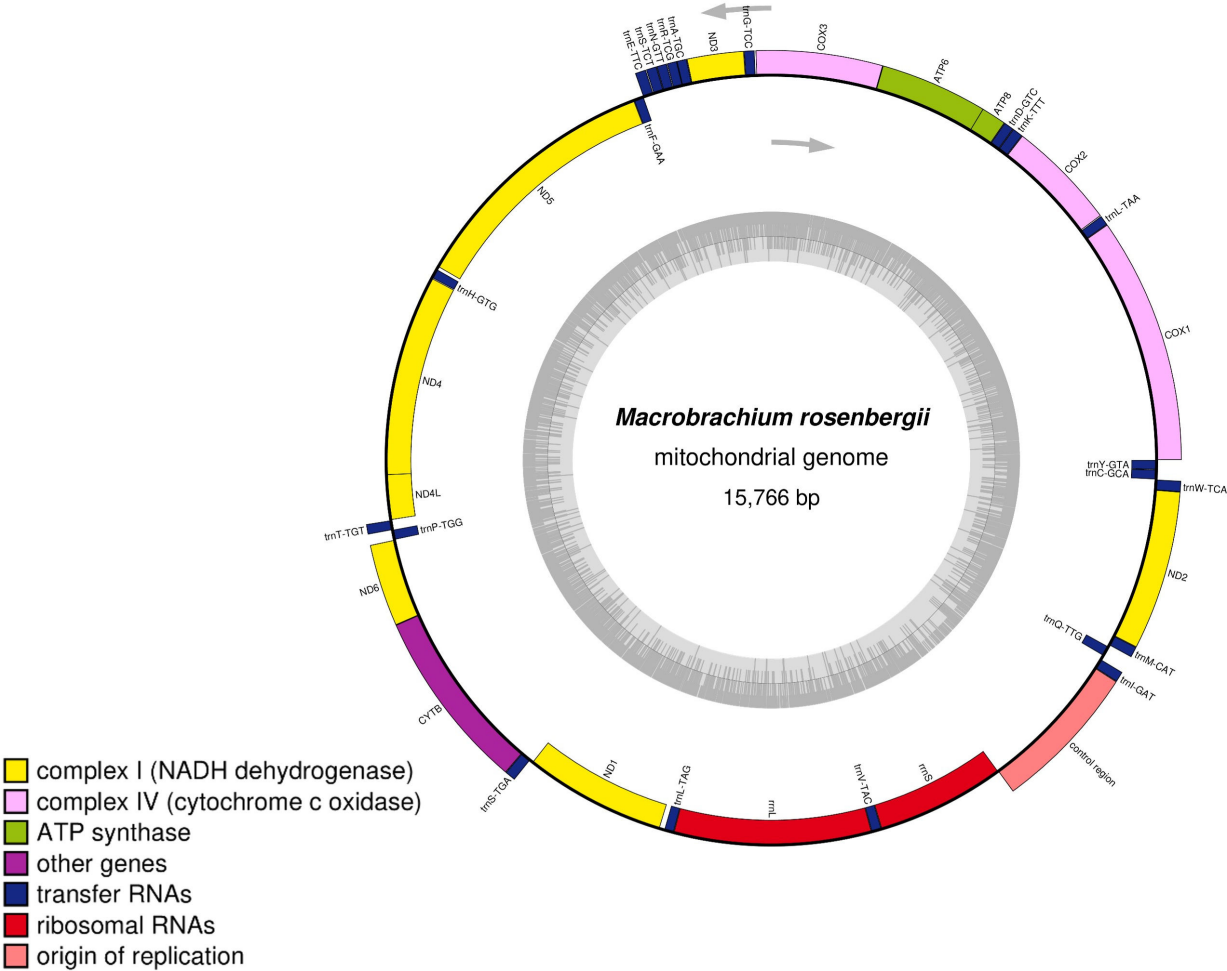
The complete mitochondrial genome map of *Macrobrachium rosenbergii*. The innermost ring illustrates the GC content, shown in ash gray, with transcription directions indicated by arrows. The outermost ring displays the genes, with those transcribed clockwise placed on the inside of the circle and those transcribed counterclockwise on the outside. The color coding corresponds to different gene groups, as indicated in the key located in the bottom left corner. The annotated map includes 13 protein-coding genes, 2 ribosomal RNA genes (*rrnS* for 12S ribosomal RNA and *rrnL* for 16S ribosomal RNA), 22 transfer RNA genes, and the putative control region. The genome was visualized using OrganellarGenomeDRAW.

The assembled complete mitogenome of *M. rosenbergii* is a circular sequence of 15,766 base pairs, with a G + C content of 38.1%. Our annotation identified 1 control region (D-loop), 2 rRNA-coding regions (12S and 16S rRNAs), 22 tRNAs, and 13 protein-coding genes (Fig. 1). The heavy strand encodes 23 genes, while the light strand encodes 14 genes (Table 1). The nucleotide composition of the heavy strand is as follows: 5,612 A (35.60%), 4,144 T (26.28%), 3,873 C (24.57%), and 2,137 G (13.55%). The ATP6, ATP8, COI-III, Cytb, ND2, ND3, and ND6 genes are encoded by the heavy strand, while the light strand encodes ND1, ND4, ND4L, and ND5. The circular mitomap was visualized using OrganellarGenomeDRAW (OGDRAW) (12).

**TABLE 1:** Mitochondrial genome content, organization, and codon information of *M. rosenbergii*

Gene	Location	Gene length (bp)	Start codon	Stop codon	Anti-codon	H/L strand^*b*^	Intergenic region length (bp)
cox1	1–1,535	1,535	ACG	TA^*a*^		+	0
trnL	1,536–1,599	64			TAA	+	0
cox2	1,603–2,290	688	ATG	T^*a*^		+	3
trnK	2,291–2,358	68			TTT	+	0
trnD	2,359–2,423	65			GTC	+	0
atp8	2,424–2,582	159	ATC	TAA		+	0
atp6	2,576–3,250	675	ATG	TAA		+	−7
cox3	3,250–4,038	789	ATG	TAA		+	−1
trnG	4,045–4,109	65			TCC	+	6
nad3	4,110–4,463	354	ATG	TAA		+	0
trnA	4,464–4,526	63			TGC	+	0
trnR	4,526–4,587	62			TCG	+	−1
trnN	4,589–4,653	65			GTT	+	1
trnS	4,654–4,720	67			TCT	+	0
trnE	4,723–4,791	69			TTC	+	2
trnF	4,791–4,856	66			GAA	−	−1
nad5	4,857–6,563	1,707	ATG	TAA		−	0
trnH	6,582–6,645	64			GTG	−	18
nad4	6,648–7,982	1,335	ATG	TAA		−	2
nad4l	7,976–8,275	300	ATG	TAA		−	−7
trnT	8,278–8,341	64			TGT	+	2
trnP	8,341–8,406	66			TGG	−	−1
nad6	8,408–8,923	516	ATC	TAA		+	1
cob/cytb	8,923–10,057	1,135	ATG	T^*a*^		+	−1
trnS	10,058–10,126	69			TGA	+	0
nad1	10,147–11,085	939	ATA	TAG		−	20
trnL	11,118–11,181	64			TAG	−	32
rrnL	11,182–12,485	1,304				−	0
trnV	12,486–12,552	67			TAC	−	0
rrnS	12,553–13,403	851				−	0
D-loop	13,404–14,333	930				+	0
trnI	14,334–14,400	67			GAT	+	0
trnQ	14,429–14,496	68			TTG	−	28
trnM	14,508–14,575	68			CAT	+	11
nad2	14,576–15,571	996	ATT	TAA		+	0
trnW	15,570–15,638	69			TCA	+	−2
trnC	15,640–15,703	64			GCA	−	1
trnY	15,704–15,766	63			GTA	−	0

^*a*^ Truncated termination codon.

^*b*^ Heavy (H) strand = “+”, Light (L) strand = “-”.

## Data Availability

The complete mitochondrial genome sequence of *Macrobrachium rosenbergii* has been deposited in the DDBJ/ENA/GenBank database under the accession number PQ213808. The version described in this manuscript is the first version, PQ213808.1. The raw sequencing data from this study have been deposited in the NCBI Sequence Read Archive (SRA) under the accession number SRR30436367 (BioProject accession number PRJNA1153522 and BioSample accession number SAMN43395988).
